# Combining vision and range sensors for AMCL localization in corridor environments with rectangular signs

**DOI:** 10.3389/frobt.2025.1652251

**Published:** 2025-09-05

**Authors:** Paloma de la Puente, Germán Vega-Martínez, Patricia Javierre, Javier Laserna, Elena Martin-Arias

**Affiliations:** Centre for Automation and Robotics (UPM-CSIC), Universidad Politécnica de Madrid, Madrid, Spain

**Keywords:** localization, mobile robotics, particle filter, range-sensors, markers, vision

## Abstract

Localization is widely recognized as a fundamental problem in mobile robotics. Even though robust localization methods do exist for many applications, it is difficult for them to succeed in complex environments and challenging situations. In particular, corridor-like environments present important issues for traditional range-based methods. The main contribution of this paper is the integration of new observation models into the popular AMCL ROS node, considering visual features obtained from the detection of rectangular landmarks. Visual rectangles are distinctive elements which are very common in man-made environments and should be detected and recognized in a robust manner. This hybrid approach is developed and evaluated both for the combination of an omnidirectional camera and a laser sensor (using artificial markers) and for RGB-D sensors (using natural rectangular features). For the latter, this work also introduces RIDGE, a novel algorithm for detecting projected quadrilaterals representing rectangles in images. Simulations and real world experiments are presented for both cases. As shown and discussed in the article, the proposed approach provides significant advantages for specific conditions and common scenarios such as long straight corridors.

## 1 Introduction

The localization problem is defined as the estimation of the robot or sensor pose within a certain environment (Thrun et al., 2005). Localization performance is considered a key aspect for autonomous mobile robots to perform high-level tasks (Hornung et al., 2014).

The most successful localization methods are probabilistic approaches, and the most popular ones are based on Markov Localization (Thrun et al., 2005). In particular, Monte Carlo Localization (MCL) is a widely known method which represents the probability density as a set of samples. The main advantages of MCL are that it can handle non-gaussian noise in sensor readings, it is multi-hypotheses, it is easy to implement, and it can adapt to the available computational resources by controlling the number of samples in its adaptive version, most commonly referred to as AMCL (Dellaert et al., 1999). Also, they usually work on grid-based maps, especially convenient for planning and navigation in unstructured environments.

In practice, the estimation results of AMCL strongly depend on the conditions of the environment, the robot and the available sensors. Using only one type of exteroceptive sensor can diminish the quality of the estimations due to a lack of recognizable features for a particular sensor setup and modality. Furthermore, using only one type of environment representation also presents limitations (Wurm et al., 2010).

Scan-based localization is not a good option in the absence of geometric landmarks (Nobili and Tinchev, 2018) or when reflective surfaces are found (Koch et al., 2017). On the other hand, vision-based localization generates less smooth results (Houben et al., 2016) and may be sensitive to illumination changes (Mondejar-Guerra et al., 2018). Using artificial landmarks may help (Kalaitzakis et al., 2021), but it involves environment modifications, highly undesirable in many real-world applications (Farkas et al., 2012).

Indeed, a common problem in robotics projects is found in corridors and similar environments, since there is a lack of geometric features for scan-based methods (de la Puente et al., 2019; Shao et al., 2019; Zeng et al., 2020; Zhang and Maher Atia, 2020; Ge et al., 2021; Peña-Narvaez et al., 2023). This kind of environment, which could be modeled as four straight walls in the shape of a rectangle (de la Puente and Rodriguez-Losada, 2015), is prevalent in offices, hospitals and industrial settings, for example, posing a challenge to robots that solely rely on geometry recognition.

The proposed solution is to augment the range-based algorithm with visual information about the environment, by means of pre-known rectangular markers that can help differentiate geometrically similar spaces. Rectangular elements are prevalent in man-made environments and offer greater distinctiveness than points or lines. Hence, the hybrid method we present enhances versatility and robustness, with significant practical applicability. The corridor environment is the most typical and relevant case, but the method also presents advantages in wide semi-open spaces where range sensors do not detect any objects. If there are obstacles in the corridor that can be detected by the range sensor, then standard AMCL should work well.

We incorporate particular options for artificial markers detection with omnidirectional cameras and a novel algorithm for natural rectangles detection in RGB cameras, but the implementation is modular and those components could be easily replaced by alternative ones.

This article is an extension of our previous work (Javierre et al., 2019; Vega-Martínez et al., 2024). Besides offering an integrated view, we now include a more complete survey of related works, additional figures and more experiments and results. New reported results include examples of rectangle detection in real dataset images as well as quantitative results of the omnidirectional vision with artificial markers approach.

## 2 Related work

### 2.1 Artificial markers detection

Many applications can benefit from the incorporation of fiducial markers as unique landmarks that are easier to detect and recognize than natural landmarks (Shibata and Yamamoto, 2014; Kirsch et al., 2023). Popular choices are AprilTag (Wang and Olson, 2016), ArUco (Romero-Ramirez et al., 2018) and STag (Benligiray et al., 2019), which have been recently compared in Kalaitzakis et al. (2021).

Another novel option is MoiréTag, which provides full 6D tracking together with camera intrinsics estimation, with improved angular accuracy (Qiu et al., 2023). DeepTag is another relevant option which supports existing markers and simplifies the design and detection of new marker patterns (Zhang et al., 2023) another interesting comparative study has been recently published (Jurado-Rodriguez et al., 2023).

### 2.2 Rectangle detection in RGB images

The problem of detecting rectangular shapes in 3D space from their 2D projections in images is a relatively underexplored question in computer vision. Previous works on the topic already indicated the potential benefits of developing these techniques for robot localization and mapping (Shaw and Barnes, 2006), for panel recognition (Wu et al., 2011), for car license plate identification (Li, 2014; Xie et al., 2018) and for target detection and tracking of spacecraft (Huadong and Yang, 2015). Other application cases include grasp detection (Karaoguz and Jensfelt, 2019) and building contour extraction (Elouedi et al., 2012).

One of the most interesting implementations is a rule-based algorithm that has not been formally published but received a demo award and shows nice results in real time videos (Shibata and Wu, 2016). Apple’s Vision Framework^1^ also highlights the relevance of the problem, addressing it by means of different rule-based strategies with several configuration options.

A possible way to approach rectangle detection is to use neural networks that detect objects with points instead of standard bounding boxes, following the idea of CenterNet (Zhou X. et al., 2019), a method originally developed for human pose detection. However, the scarcity of public datasets for this task make it difficult to train the network.

As related problems, end-to-end detection of wireframes (Huang et al., 2018; Zhou Y. et al., 2019), polygons (Wei et al., 2024) and cuboids (Liu Q. et al., 2024) have also received significant attention recently.

### 2.3 Hybrid localization approaches combining sensing modalities

Adaptive MCL based on occupancy grid maps for laser sensors is widely adopted in many robotics projects. The main reasons are related to its versatility for non structured environments (Sprunk et al., 2017), to its robustness and also to the availability of an open-source implementation integrated in ROS. Adaptation to RGB-D sensors by means of a virtual 2D laser projection is quite common too (de la Puente et al., 2019). As an alternative, efficient and reliable methods for visual localization exist as well. It is worth noting two variants of the MCL algorithm for omnidirectional cameras. In Menegatti et al. (2006) the distances to the closest color transitions are used while in Andreasson et al. (2005) no motion model is considered and the ceiling lights are recognized as reliable features.

Hybrid methods are developed so as to increase the reliability and effectiveness of localization algorithms. Fusing data from several sensors helps obtain a proper pose estimate in particularly difficult conditions (Houben et al., 2016; Zaffar et al., 2018).

The specific case of merging information from an omnidirectional camera and a laser sensor has been previously explored using low level visual cues and an EKF algorithm (Hoang et al., 2013), with promising results in a long urban trajectory. The integration of data from a lidar and a 360-degree camera has recently been addressed to obtain wide field-of-view coloured point clouds, to be used in robot navigation or scene recontruction tasks (Liu B. et al., 2024).

Combining information from a monocular camera and a lidar sensor has been proposed before for resolving ambiguity problems related to symmetries in the environment (Ge et al., 2021). In this case, the motivation is similar to ours and a particle filter is employed, but this work obtains ORB features instead of rectangles, and they are only integrated when a symmetry situation is detected. The matching process of low level visual features faces important similarity challenges that can cause recognition and association failures (Javed and Kim, 2022). This risk can be greatly diminished when integrating rectangular distinctive elements as we propose.

### 2.4 Advanced localization with particle filters

Particle filters present relevant advantages such as robustness against data association errors and suitability for unstructured scenarios. Hence, different observation models based on traditional geometry or learning-based methods have been developed in the last years.

One of the first related approaches that inspired our work proposed the integration of visual lines for localization of humanoid robots, particularly for climbing stairs (Hornung, 2014). Other pertinent work focuses on MCL localization by means of text spotting, developing two different observation models (Zimmerman et al., 2022). Like ours, this approach does not entail environment modifications, but it depends on the presence of such textual cues in the scene and requires text recognition algorithms. Another recent article integrated semantic information, presenting a new advanced method for particle generation (Peña-Narvaez et al., 2023).

Applying deep learning to the particle filter localization paradigm has yielded promising results as well (Karkus et al., 2018; Chen et al., 2020). Other contributions focus on robustness (Eder et al., 2022) and failure detection, improving localization recovery times (Akai, 2023; García et al., 2023).

## 3 Methodology

### 3.1 Artificial markers detection using an omnidirectional camera

In our implementation, the visual markers are specifically designed with a configuration of 5 rows and 5 columns, typically printed in A3 size, to facilitate robust identification and pose estimation within the hybrid AMCL localization framework. The first and last rows remain invariant, while the other ones contain binary information that changes depending on the marker location. Specifically, *map ID* determines the building where the marker is placed, *sector ID* depends on the marker room, and *marker ID* provides a unique identifier for that particular landmark inside that room. The visual markers coordinate system is placed at the center of the rectangle, with the z-axis pointing forward.

For their detection and recognition, the algorithm developed in Alvarado (2017) was integrated into our ROS-based system. The four corners of each marker are independently found by a method applying contour detection. Possible errors in the detection of one of these points can be balanced by the properly determined ones.

### 3.2 Natural rectangles detection using an RGB-D camera

**FIGURE 1:**
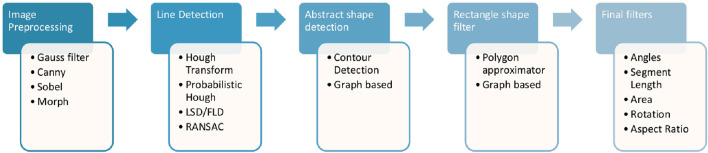
Typical components of a rectangle detection method.

A traditional approach to rectangle detection would follow a process with five key components, as shown in Figure 1. The initial step is about pre-processing the image, typically including grayscale conversion and the application of a Gaussian filter to mitigate noise. Subsequently, a line detector, often based on the Hough transform, is used to extract all lines from the image. The following step extracts shapes from lines, with methods such as contour detection or graph-based techniques. Finally, rectangles are isolated based on the number of sides, followed by the application of a series of filters to reject false or irrelevant detections.

The output of such a procedure, however, presents limited accuracy and reliability. The line extraction results have a strong influence on the rectangle detection, and shape extraction usually creates polygons with more than four sides that must be removed. Also, there are significant challenges related to partially obscured rectangles and missing detections when the detected segments are slightly shorter than the real ones.

To overcome the identified limitations, we propose RIDGE (Rectangle Intersection-based Detector using Graphs and Elongation), a novel algorithm designed for robust rectangle detection. RIDGE is grounded in the principle that polygon vertices correspond precisely to the intersection points of their defining line segments. The algorithm initially leverages the Fast Line Detection (FLD) method (Lee et al., 2014) to extract candidate line segments from the image.

FLD, as implemented in OpenCV, begins by applying the Canny Edge Detector to isolate edges. It then employs Pixel Chaining to find pixel chains that align in any direction, iterating over all non-zero pixels. Following this, Segment Filtering and Operations are performed to filter chains based on their length and proximity to the image border, and calculate the angle and endpoints for the remaining segments. An optional Segment Merging step can merge segments that are approximate continuations of one another. However, FLD may generate multiple, misaligned segments for a single line, leading to incomplete and jagged sides in the detection process, and consequently, the polygon approximator often produces shapes with more than four sides, challenging the accurate detection of rectangles by traditional methods. In RIDGE, the resulting segments are elongated to bridge minor gaps and compensate for imperfections, ensuring that genuine intersections are not overlooked. Enlarged segments are drawn in a “lines image”, in white colour over black background, for subsequent line validation.

The intersection extractor systematically goes through all the detected segments, identifying intersections among them using the algorithm outlined in Goldman (1990). This algorithm treats the segments as vectors and seeks the fraction of each vector that corresponds to the intersection. To increase computational efficiency and accuracy in scenarios where multiple intersections may correspond to a single corner, Non-Maximum Suppression (NMS) is employed to reduce overlapping detections.

RIDGE then creates a graph structure by connecting these intersection points (vertices), provided the corresponding elongated segments generating them exhibit sufficient support, represented by white pixels, in the FLD-generated segment image. Finally, the graph is traversed systematically to identify closed paths consisting of exactly four connected segments, effectively isolating rectangular shapes.

**FIGURE 2:**
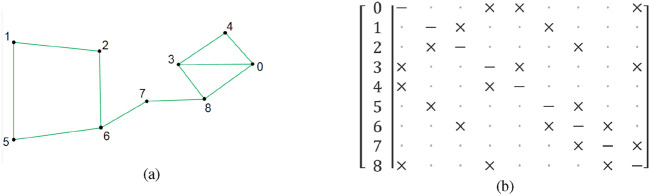
**(a)** Example graph. **(b)** Corresponding adjacency matrix, × represents valid connections (Reprinted with permission from Vega-Martínez et al. (2024). Copyright © 2024, IEEE).

A symmetric adjacency matrix of Boolean values is used to represent the graph. Rows and columns correspond to corners, and elements are set to True if there is a valid line connecting them. The diagonal elements of the matrix must all be False, since they would indicate the connection of a corner with itself (see Figure 2).

**FIGURE 3:**
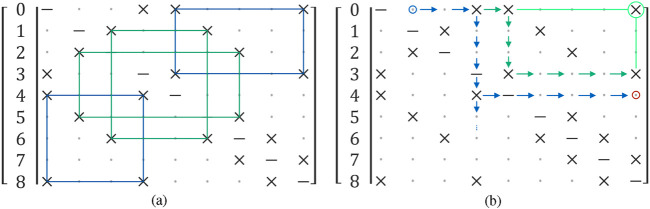
**(a)** Rectangles present in the adjacency matrix, rectangles with the same colour represent the same rectangle in the graph. **(b)** Traversal order for searching rectangles, the blue circle is the starting point, the red circle represents an end of path without detection, and the green circle with lines represents the last check for a valid connection that forms a rectangle (Reprinted with permission from Vega-Martínez et al. (2024). Copyright © 2024, IEEE).

The current graph encompasses all polygons, and the subsequent objective is to isolate quadrilaterals from other shapes. In the context of RIDGE, a rectangle is identified as a closed path consisting of four consecutive segments that share common endpoints, effectively forming a loop. Within the adjacency matrix structure (see Figure 3a), this pattern emerges when connections alternate between rows and columns corresponding to shared corners. The symmetry of the matrix simplifies the search process, requiring only traversal of its upper triangular part. Upon locating an initial link, the algorithm switches orientation orthogonally to detect a second segment and crosses the diagonal to continue the sequence. It then returns to the original direction to locate the third connection and ultimately verifies whether the final segment completes the quadrilateral (see Figure 3b). This logic ensures that the corners define a closed rectangular path. Furthermore, RIDGE is capable of identifying more complex polygonal shapes by extending the traversal pattern, including support for wrapping paths and special handling of diagonally indexed entries for polygons with an odd number of sides.

The last stage in RIDGE is the application of filters to minimize false positives:

- Segment Length Consistency: Rectangular candidates are required to have sides of comparable lengths; large discrepancies among segment lengths trigger rejection to eliminate distorted shapes.
- Area and Shape Proportion: Elements whose area is disproportionately small or large relative to the full image are discarded using a predefined area-to-image ratio. Furthermore, shape regularity is evaluated using the Polsby-Popper metric, which contrasts perimeter and area to reject irregular or elongated contours.
- Angular Deviation: To avoid misclassifying configurations with adjacent nearly parallel sides, the algorithm verifies that internal angles lie within an acceptable band around 90°, defined as π/2±a , with a serving as the tolerance margin.
- Convex Shape Verification: The candidate is also tested for convexity to ensure that inward-pointing vertices, typical of star or dart-like figures, are eliminated.

**FIGURE 4:**
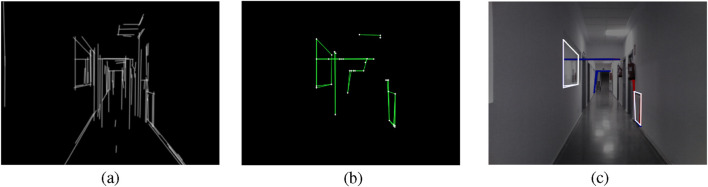
RIDGE detection example: **(a)** FLD result with elongated lines, **(b)** Graph of lines and corners, **(c)** Extracted rectangles, white rectangles are valid, red ones are discarded by the length test, blue ones are discarded by the area test, and yellow ones are discarded by the convexity test (Reprinted with permission from Vega-Martínez et al. (2024). Copyright © 2024, IEEE).

**FIGURE 5:**
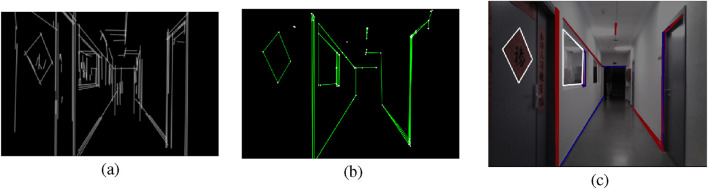
RIDGE detection example. **(a)** FLD result with elongated lines, **(b)** Graph of lines and corners, **(c)** Extracted rectangles, white rectangles are valid, red ones are discarded by the length test, blue ones are discarded by the area test, and yellow ones are discarded by the convexity test.

RIDGE detection examples corresponding to the real corridor of Section 5.2.2 are depicted in Figures 4, 5.

Once rectangles have been robustly detected using RIDGE, the next step is to incorporate these observations into the particle filter framework. To this end, we extend the standard AMCL algorithm to support visual cues, giving rise to the Hybrid AMCL formulation described below.

### 3.3 Observation model for rectangular features

The observation model integrated into Hybrid AMCL evaluates how well the corners of a detected rectangular feature align with the projected corners of a known marker, as viewed from the hypothetical pose defined by each particle. Since the detection is assumed to originate from the robot’s actual position, this comparison provides a metric for assessing the plausibility of each particle’s estimated pose.

In the first place, landmark projection requires the pose of the map rectangle with respect to the camera. For each particle and each rectangle considered, the relative pose of the four corners is calculated by assuming that the robot pose is the one represented by the sample.

**FIGURE 6:**
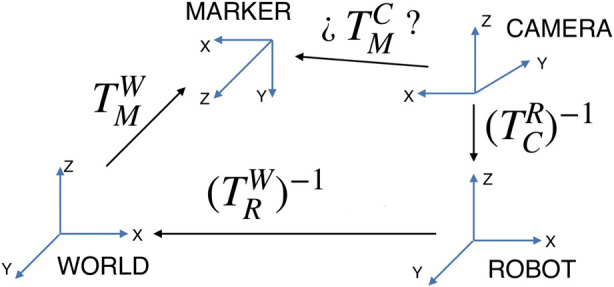
Relative transformations to obtain the map marker pose with respect to the camera.

The transformation from the world frame to the rectangular marker frame is denoted $\left(T_{M}^{W}\right)$ , while the transformation from the world to the robot base coordinates is given by $\left(T_{R}^{W}\right)$ and the one from the robot base to the camera frame is $\left(T_{C}^{R}\right)$ , as shown in Figure 6.

The $\left(T_{M}^{W}\right)$ transformation for each rectangle is known from the landmarks map, $\left(T_{C}^{R}\right)$ is obtained from the robot’s setup, and $\left(T_{R}^{W}\right)$ is defined by the particle. The unknown transform $\left(T_{M}^{C}\right)$ can be obtained as $T_{M}^{C}={\left(T_{C}^{R}\right)}^{-1}\cdot {\left(T_{R}^{W}\right)}^{-1}\cdot \left(T_{M}^{W}\right)$ , or by directly traversing the transform tree, operating with the ROS TF package^2^.

Afterwards, this result is used to project the corners onto the corresponding image, based on the camera model and intrinsic parameters. The observation model could be adjusted to use any camera model by swapping the projection module.

In the case of an omnidirectional camera, the projection is obtained by using CMei’s model (Mei and Rives, 2007) and *omnidir* module of OpenCV^3^. In the case of an RGB camera, the projection is obtained by means of the pinhole camera model, as the detector uses rectified images. This provides the position of the map rectangles’ corners in pixel coordinates if the robot were in the position of the considered sample.

To assess how closely the observed rectangle $\left(x_{d,i},y_{d,i}\right)$ matches the expected projection $\left(x_{p,i},y_{p,i}\right)$ , a normalized Euclidean distance is computed for each corresponding pair. The differences in coordinates are divided by the image’s width and height to ensure the metric is invariant to resolution and sensor characteristics. The total discrepancy is then calculated as the cumulative distance over all four corners, as shown in Equation 1.

$$L_{m}=\overset{4}{\underset{i=1}{\sum }}\sqrt{{\left(\frac{x_{d,i}-x_{p,i}}{width}\right)}^{2}+{\left(\frac{y_{d,i}-y_{p,i}}{height}\right)}^{2}}$$ (1)

Instead of adopting a Gaussian likelihood function, the model defines the observation probability using an exponential decay based on the computed alignment error, in line with the formulation proposed in Hornung (2014). Outlier detections with large errors are directly discarded via a thresholding mechanism, avoiding explicit random measurement modeling. The resulting expression is given in Equation 2.

$$p\left(z_{t}|x_{t}^{\left[m\right]},m\right)=\underset{i\in \left\{det\right\}}{\sum }z_{\mathrm{hit}}\cdot \lambda e^{-\lambda \cdot L_{m}}$$ (2)

Each particle’s likelihood is adjusted according to the standard structure of the Likelihood Field Model implemented in the ROS version of AMCL. The constants $z_{\mathrm{hit}}$ and λ serve as tunable hyperparameters that govern the sensitivity of the visual observation model.

When dealing with naturally occurring rectangular features, the correspondence module evaluates each detection against all markers that would be visible from the pose hypothesis of a given particle. Among the candidate projections, the one that results in the minimum alignment error is selected, according to the metric previously defined. Since detections and projections may differ in corner ordering or visibility, corner associations are resolved by iteratively matching each detected point to the closest projected corner, minimizing the total pairing error.

## 4 RIDGE detection results

**FIGURE 7:**
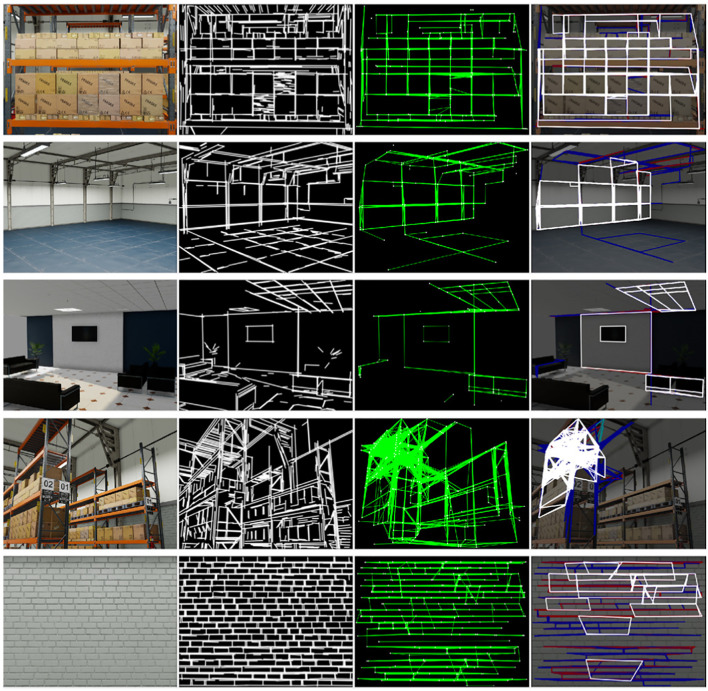
RIDGE detection examples for images from Isaac Sim. First column: original image; Second column: FLD result with elongated lines; Third column: Graph of lines and corners; Fourth column: extracted rectangles, only white rectangles are valid (Reprinted with permission from Vega-Martínez et al. (2024). Copyright © 2024, IEEE).

A series of test images from various photorealistic scenarios derived from Isaac Sim^4^ were used to perform a qualitative analysis (see Figure 7).

The first test case corresponds to a logistics scene featuring multiple stacked boxes, selected for its simplicity in evaluating the RIDGE algorithm. The detector performs well overall, correctly identifying most of the rectangular surfaces. However, some plastic-wrapped boxes introduce spurious edges during the FLD stage, complicating the resulting graph and leading to the rejection of certain rectangles.

Similarly, the second and third scenes yield accurate detections. It should be noted that the algorithm does not detect floor tiles, which is attributed to the limited sensitivity of FLD in such low-contrast patterns.

The benefits of the segment elongation strategy are illustrated in the living room setup. Although the ceiling lamp segments are visible in the line image, the failure to build a corresponding graph suggests that the FLD-generated lines were too short to establish meaningful intersections.

In contrast, the final two examples expose a key limitation of the method: scenes with heavy texture. In these cases, RIDGE produces overly dense graphs that include many false positives, which not only degrade detection accuracy but also increase computational burden.

Another series of tests were conducted on real images from the Pare scenario in the Robot@Home dataset (Ruiz-Sarmiento et al., 2017, Figure 8).

**FIGURE 8:**
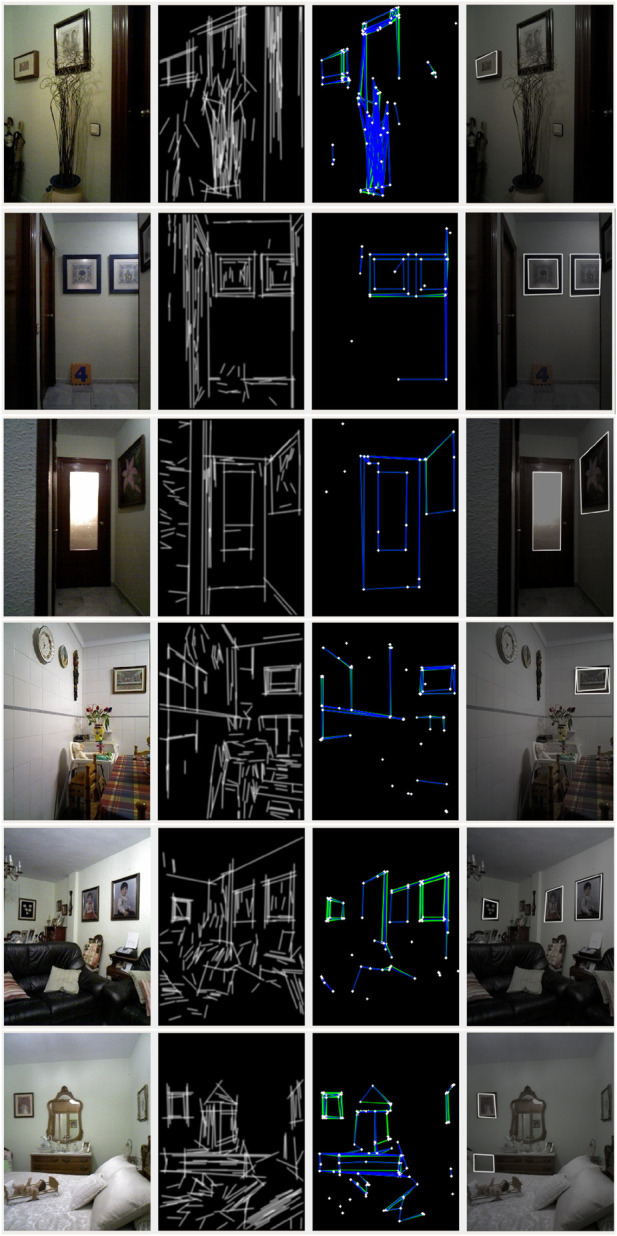
RIDGE detection examples for real images from the Robot@Home dataset (Ruiz-Sarmiento et al, 2017).

In the first image, the small picture on the wall is correctly detected, while the plant generates occlusions that prevent the bottom line of the largest picture from being extracted. This is the expected result, occlusions are hard to handle. The second row of images shows a nice detection of the pictures, including both their inner and outer countours. The partial occlusion of the picture on the right could cause problems to the matching algorithm, which should be carefully configured.

The picture on the wall of the third picture is nicely detected. The inner contour of the door is also well selected, while the door itself is not detected because FLD line extraction includes false additional lines on the bottom part.

The detection of the picture from the fourth image is not very accurate and there are redundant overlapping detections, but this should not affect the localization process if the expected covariance values of the rectangle detection process are properly assigned.

The fifth image presents a nice detection of pictures in the living room of the Pare apartment. The last image includes a correct detection of the picture on the wall and a false positive detection related to the elongation of lines in a particular setting with aligned furniture pieces.

Overall, most of the problems occur due to excessive detections of lines. Too aggressive elongations bring about false positive detections in a few cases, as well.

**FIGURE 9:**
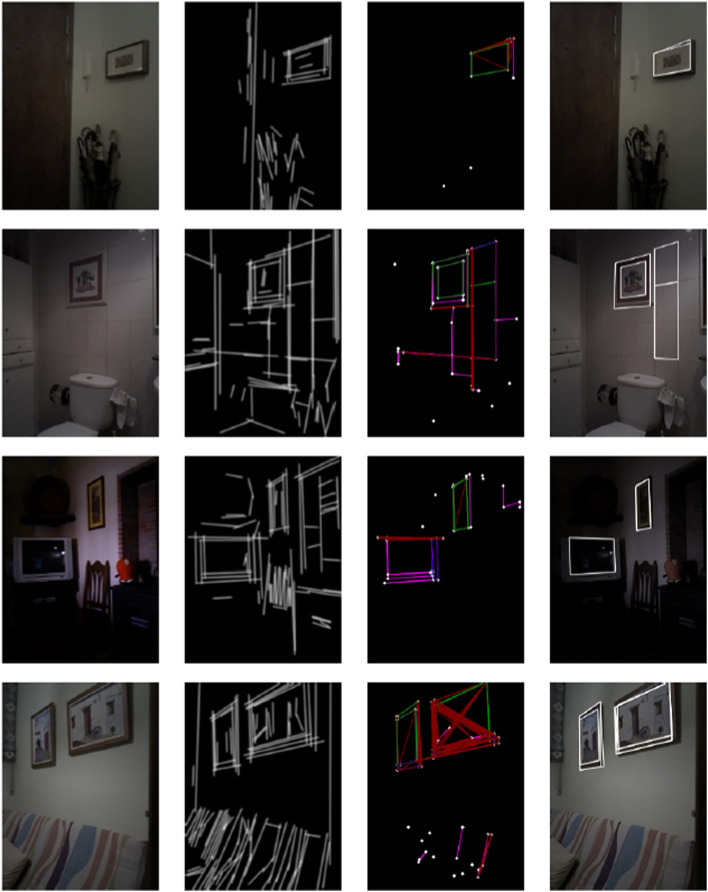
RIDGE detection examples for real images from the Robot@Home dataset (Ruiz-Sarmiento et al., 2017), using Airline for line detection instead of FLD.

We have also tested RIDGE based on the Airline learning-based method for line extraction (Lin and Wang, 2023). This method is more robust and the extracted lines are thicker, which requires RIDGE parameter tuning to get improved detections. In particular, the NMS threshold should be increased, to reduce the number of overlapping rectangles. Figure 9 shows some examples of results.

It is worth noting that the detector results are important but should not be critical, since the map only contains selected rectangles of interest and the particle filter approach presents significant robustness against data association errors (Dong et al., 2023).

## 5 Localization results

Corridor-like environments pose a particular challenge for standard AMCL due to their lack of geometric features and considerable length, which often exceeds the LiDAR’s effective range. For this reason, the experimental validation focuses on these scenarios. The hallway maps used during testing are oriented along the X axis, which hence concentrates the majority of the accumulated uncertainty and drift in the estimation. Conversely, the Y and θ components are more stable, as LiDAR data can continually correct them with minimal error accumulation, by detecting the lateral walls.

To quantify localization performance, the Absolute Positioning Error (APE) is employed as the evaluation metric. Given the nature of the environments under study, only the X -axis error is considered in the analysis (see Equation 3).

$$APE_{x_{i}}=|x_{\mathrm{est}}-x_{gt}{|}_{i}$$ (3)

For a fair comparison, rosbag files were recorded so that each version of the algorithm was tested with the same data. It should be noted that, given the stochastic nature of the AMCL algorithm, there is an inherent variability in the results across different runs.

### 5.1 Localization based on artificial markers detection using an omnidirectional camera

#### 5.1.1 Simulation experiments

**FIGURE 10:**
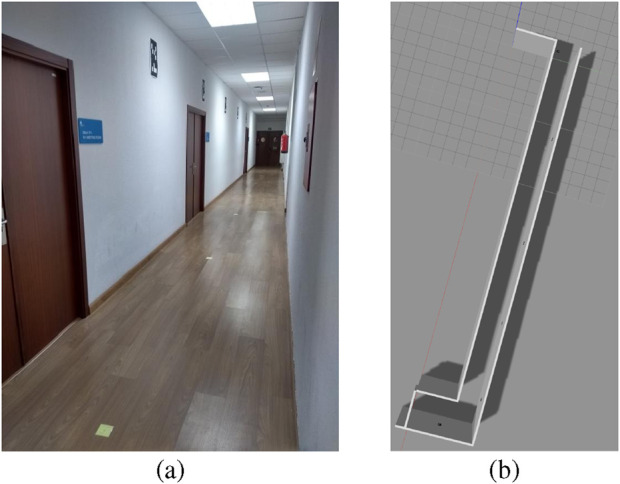
**(a)** Real corridor and **(b)** Gazebo simulation environment for artificial markers-based hybrid localization.

We present results from a simulated environment based on a real university environment, as shown in Figure 10. This is a corridor 25.6 m long and 1.66 m wide. Five markers were placed on one of the walls, with a separation distance equal to 3 m. The simulations were developed using Gazebo in a ROS-based project.

**FIGURE 11:**
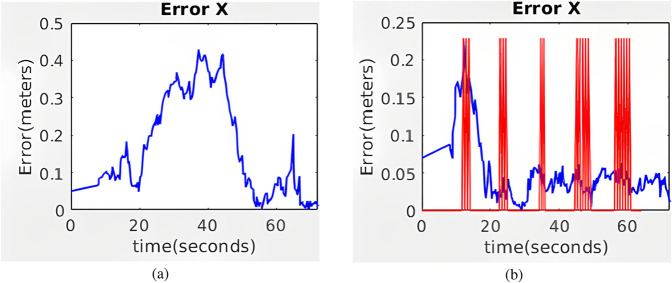
Evolution of the absolute value of the error along the simulated corridor with five artificial markers for **(a)** AMCL and **(b)** Hybrid AMCL. The red vertical lines indicate marker detections.

The evolution of $APE_{x}$ along the corridor is shown in Figure 11 for standard AMCL and hybrid AMCL. As expected, with standard AMCL the absolute position error grows significantly along the corridor direction. When using hybrid AMCL, the detection of the markers allows for an important error reduction along the corridor.

#### 5.1.2 Real experiments

The robot that was used for these experiments is a Patrolbot robot platform, no longer commercialized. The camera is an omnidirectional camera SONY RPU-C3522, which provides a 360° field of view with a CCD image sensor. It has a frame rate of 7.5 Fps and its dimensions are 75 × 66.5 × 75 mm without lens. The camera was added using a methacrylate structure at a height equal to 1.4 m.

In the real experiments, an accurate measurement of the groundtruth position given by external devices was not available. The groundtruth was estimated by means of measured markers on the floor, in a stop-and-go manner, applying linear interpolation assuming constant speed between the markers.

**FIGURE 12:**
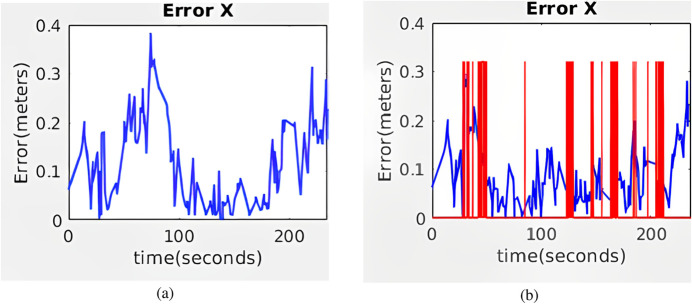
Evolution of the absolute value of the error along the real corridor with five artificial markers for **(a)** AMCL and **(b)** Hybrid AMCL. The red vertical lines indicate marker detections.

The evolution of $APE_{x}$ along the corridor is shown in Figure 12 for standard AMCL and hybrid AMCL. When using the hybrid version of AMCL, the maximum error is below 0.3 m and it is quickly reduced once the first marker is detected.

**FIGURE 13:**
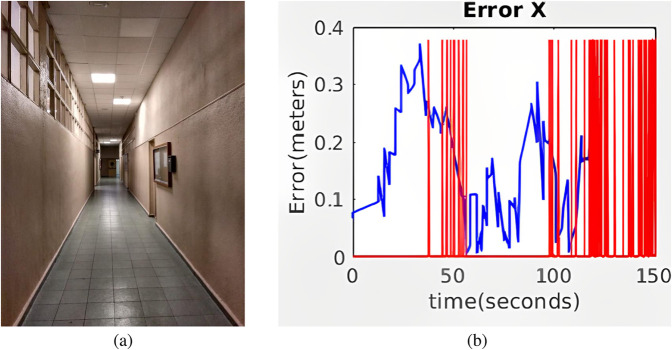
**(a)** Real corridor for real experiment 2. **(b)** Evolution of the absolute value of the error along the second real corridor with three artificial markers for Hybrid AMCL. The red vertical lines indicate marker detections.

We also present results from another experiment in a real university corridor 14.2 m long and 2.11 m wide (See Figure 13). Two artificial markers were placed on one wall, with a separation equal to 6 m, and another one was placed on the other wall.

In this experiment, the maximum $APE_{x}$ recorded with standard AMCL was 0.579 m, whereas the hybrid approach achieved a lower maximum of 0.371 m. The Absolute Trajectory Error (ATE) for standard AMCL was 0.285 m, while for hybrid AMCL it was 0.166 m.

**FIGURE 14:**
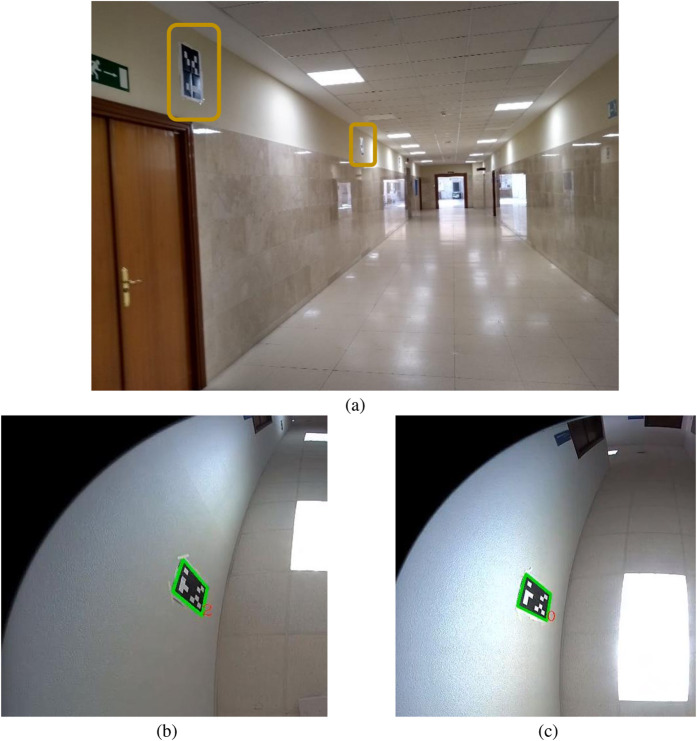
**(a)** Illumination challenges in a corridor scenario. **(b)** Correct detection of marker number 2. **(c)** Incorrect recognizion of marker number 4.

Other real corridor environments presented serious illumination challenges that caused problems to the artificial markers detector, as shown in Figure 14.

### 5.2 Localization based on natural markers detection using an RGB-D camera

#### 5.2.1 Simulation experiments

To evaluate the robustness of Hybrid AMCL under conditions where conventional AMCL does not work well, two different test environments were specifically designed.

##### 5.2.1.1 Long gallery test

**FIGURE 15:**
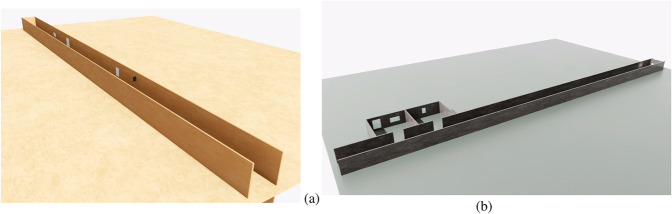
**(a)** Long Gallery environment. **(b)** Long Hallway environment (Reprinted with permission from Vega-Martínez et al. (2024). Copyright © 2024, IEEE).

**FIGURE 16:**
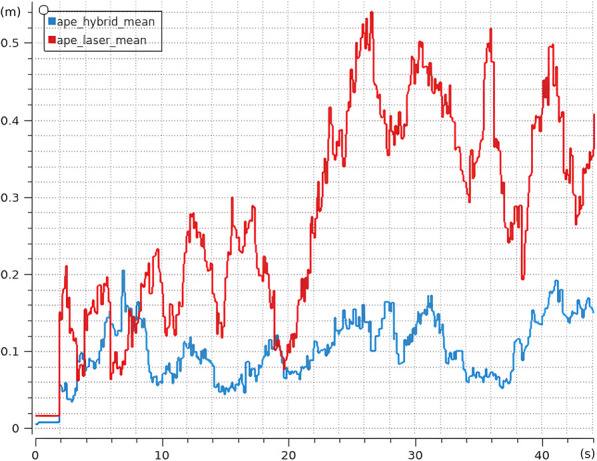
Average APE for Hybrid AMCL and AMCL over 10 runs in the long corridor environment.

The first environment, referred to as the Long Gallery (Figure 15a), consists of a 40-m corridor populated with rectangular elements such as signs or framed images distributed along the walls. This setup is specifically intended to evaluate the behavior of the system under continuous visual feedback. As shown in Figure 16, the Absolute Positioning Error (APE) for standard AMCL exhibits a steady increase over time. In contrast, Hybrid AMCL maintains low and bounded error values throughout most of the trajectory, with a noticeable increase only near the end, once the robot runs out of visible rectangles and consequently loses visual reference.

##### 5.2.1.2 Long hallway test

The second test scenario corresponds to a 40-m hallway lacking both geometric and visual landmarks (Figure 15b). At the far end, two rooms are present with identical structural layouts; however, one of them contains a single rectangular feature on the wall, while the other includes two. The primary purpose of this setup is to force ambiguity in the pose estimation by dispersing the particle cloud uniformly along the corridor, making it necessary for the robot to rely on visual cues to resolve the final location.

**FIGURE 17:**
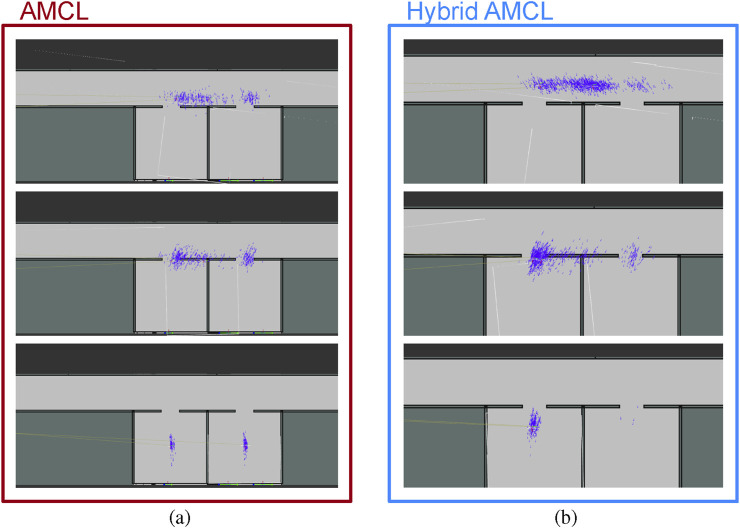
Particle cloud evolution for AMCL **(a)** and Hybrid AMCL **(b)** in the Long Hallway test (Reprinted with permission from Vega-Martínez et al. (2024). Copyright © 2024, IEEE).

Hybrid AMCL demonstrates the ability to correctly identify the intended room by leveraging visual information, whereas the standard AMCL approach tends to split the particle cloud between both possibilities, often oscillating between them or even committing to an incorrect hypothesis. This behavior is illustrated in Figure 17, highlighting the improved reliability of the hybrid method in scenarios where range-only localization leads to significant uncertainty and multimodal pose distributions.

#### 5.2.2 Real experiments

**FIGURE 18:**
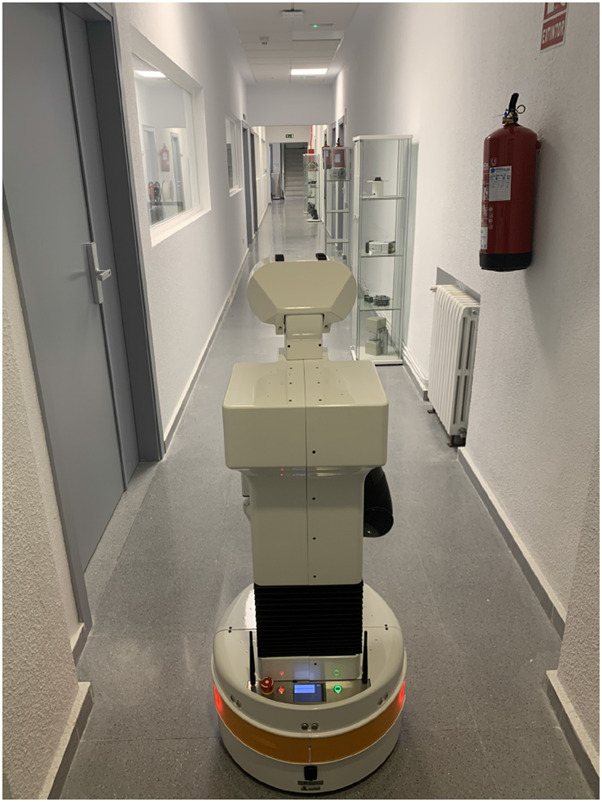
Tiago robot at the real experiment hallway.

A Tiago robot^5^ with a laser sensor with 25 m maximum range and an RGB-D camera was used for these experiments. The selected hallway for this test is approximately 30 m long and 1.6 m wide (See Figure 18). It presents few geometric references along the corridor direction while including several rectangular elements of interest for this work.

An occupancy grid of the hallway is initially constructed using the Gmapping algorithm. Nevertheless, since Gmapping, like standard AMCL, relies exclusively on LiDAR data, it struggles to accurately represent environments that lack sufficient geometric features. To overcome this limitation, a precision laser rangefinder is employed to manually measure the full length of the corridor. These measurements are then used to correct the generated map, ensuring that it accurately reflects the true physical dimensions.

**FIGURE 19:**
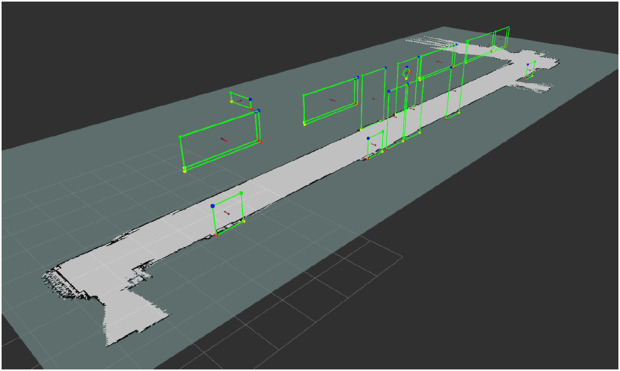
Occupancy grid and marker map of the environment for the real experiment (Reprinted with permission from Vega-Martínez et al. (2024). Copyright © 2024, IEEE).

A visual marker map was manually built. The process begins by identifying candidate rectangles in the environment, and then their exact location, width, and height are recorded using the same laser rangefinder (see Figure 19).

To obtain ground truth data for the X position, using the laser range finder, the robot is stopped at regular intervals along the hallway. During navigation, the robot advances slowly while performing small adjustments to remain centered within the corridor. At each stop, the distance to the robot’s torso is recorded, providing reference points for later evaluation. In this experiment, we opt to exclude trajectory-wide motion errors and instead focus solely on pose estimates obtained during stationary periods, which coincide with the availability of ground truth measurements.

**FIGURE 20:**
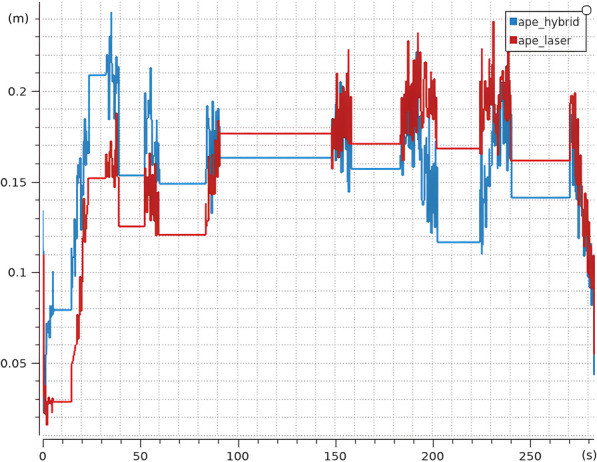
Average APE for Hybrid AMCL and AMCL over 10 runs in the real environment (Reprinted with permission from Vega-Martínez et al. (2024). Copyright © 2024, IEEE).

The results, shown in Figure 20, reveal that although AMCL begins with slightly lower error values, its accuracy degrades progressively along the hallway. In contrast, Hybrid AMCL exhibits better overall performance as it leverages visual landmarks to constrain the estimate. It is important to mention that this particular hallway is not entirely devoid of features, elements like recessed doorways and heating units provide enough structure for AMCL to make corrections, which mitigates the type of error accumulation observed in the Long Gallery test.

Nevertheless, Hybrid AMCL demonstrates its ability to enhance localization through vision-based cues. However, the accuracy of its results is inherently tied to the quality of the marker map; any significant misalignment in marker placement can introduce noticeable deviations, as seen at the beginning of the sequence.

**FIGURE 21:**
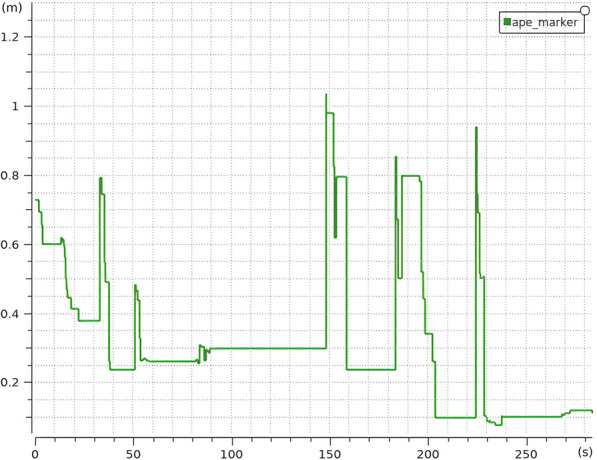
Average APE for Hybrid AMCL without LiDAR over 10 runs in the real environment (Reprinted with permission from Vega-Martínez et al. (2024). Copyright © 2024, IEEE).

Beyond the comparison between AMCL and hybrid AMCL, an additional experiment was conducted in which LiDAR data were entirely excluded from the localization process. This test was designed to evaluate whether visual rectangles alone could support reliable pose estimation. As illustrated in Figure 21, the resulting error is higher compared to the previous configurations, yet the robot remains accurately localized throughout the experiment.

These findings suggest that Hybrid AMCL retains its functionality even in the absence of range data, making it a viable option in scenarios where LiDAR is unavailable or ineffective. It should be noted that the system’s odometry remains uninitialized until the first visual rectangle is detected and incorporated into the filter, which is the reason why the Absolute Positioning Error (APE) begins with a non-zero value.

## 6 Conclusion and future work

This work presented the integration of range and vision measurements into a hybrid version of the AMCL algorithm, with the aim of solving practical problems in real robotics projects related to robot operation in corridor-like environments. Hybrid AMCL represents an enhancement over AMCL in environments with few geometric features that present rectangular visual features to be detected.

The first version of the hybrid approach integrated a laser sensor and an omnidirectional camera, and it was tested by means of artificial rectangular markers. Tests conducted in simulated and real environments showed that the error along the corridor direction is significantly reduced when artificial markers are properly detected and identified. In challenging illumination conditions, where marker IDs may not be correctly recognized, the reliability parameters of marker measurements should be re-adjusted. Since the ROS-based implementation is modular, other marker designs and detectors could also be tested.

The second version of the hybrid approach integrated a laser sensor and the camera of an RGB-D sensor. In this case, the RIDGE detector was proposed to test the approach using natural rectangular markers, eliminating the need of environment modifications. RIDGE has demonstrated effective rectangle detection capabilities, but there is still room for robustness improvements. Particularly, it presents limitations when there is a lot of texture, as FLD detects a large number of lines, which produces random corners.

The designed experiments for this version of the hybrid approach showed that, besides reducing the accumulated error along a corridor if rectangular signs are present, Hybrid AMCL proves effective in resolving ambiguity in symmetric environments, as demonstrated in the long hallway test.

Future enhancements to the RIDGE detector may involve the incorporation of other learned feature extractors, such as SuperPoint (DeTone et al., 2018), as a replacement for the current FLD-based and Airline-based segment detection methods. Another alternative would be training a dedicated neural network for quadrilateral detection directly from images. However, the lack of annotated datasets tailored to this task represents a significant obstacle. We are currently working on the generation of a synthetic dataset from an IsaacSim scenario and a labeled dataset from a selection of images from Robot@Home. Once the datasets are ready, we will focus on the development and evaluation of end-to-end learning-based approaches compared to RIDGE versions. Another interesting improvement is to combine rectangle detection with semantic detection, to filter and remove rectangles not corresponding to elements classes included in the map.

With respect to Hybrid AMCL, one avenue for improvement is the dynamic adjustment of visual marker weights based on detection confidence or quality metrics, as proposed in García et al. (2023). This could enhance robustness in cases where rectangle detections are noisy or partially occluded. Additionally, the data association process could be refined to better handle scenes with numerous overlapping or similar rectangular features. Incorporating semantic information into the recognition and matching pipeline is among the main directions currently considered.

Overall, the proposed hybrid approach broadens the applicability of AMCL in structured indoor environments by integrating visually distinctive features into the localization process.

## Data Availability

The raw data supporting the conclusions of this article will be made available by the authors, without undue reservation.

## Footnotes

1
https://developer.apple.com/documentation/vision/vndetectrectanglesrequest

2
http://wiki.ros.org/tf

3
https://docs.opencv.org/3.2.0/db/dd2/namespacecv_1_1omnidir.html

4
https://developer.nvidia.com/isaac-sim

5
https://pal-robotics.com/robots/tiago/
